# Vulnerability of Pacific salmon to invasion of northern pike (*Esox lucius*) in Southcentral Alaska

**DOI:** 10.1371/journal.pone.0254097

**Published:** 2021-07-02

**Authors:** Chase S. Jalbert, Jeffrey A. Falke, J. Andrés López, Kristine J. Dunker, Adam J. Sepulveda, Peter A. H. Westley

**Affiliations:** 1 College of Fisheries and Ocean Sciences, University of Alaska Fairbanks, Fairbanks, Alaska, United States of America; 2 U.S. Geological Survey, Alaska Cooperative Fish and Wildlife Research Unit, University of Alaska Fairbanks, Fairbanks, Alaska, United States of America; 3 College of Fisheries and Ocean Sciences, University of Alaska Fairbanks, Fairbanks, Alaska, United States of America; 4 University of Alaska Museum, Fairbanks, Alaska, United States of America; 5 Division of Sport Fish, Alaska Department of Fish and Game, State of Alaska, Anchorage, Alaska, United States of America; 6 U.S. Geological Survey, Northern Rocky Mountain Science Center, Bozeman, Montana, United States of America; USDA Forest Service, UNITED STATES

## Abstract

The relentless role of invasive species in the extinction of native biota requires predictions of ecosystem vulnerability to inform proactive management strategies. The worldwide invasion and range expansion of predatory northern pike (*Esox lucius*) has been linked to the decline of native fishes and tools are needed to predict the vulnerability of habitats to invasion over broad geographic scales. To address this need, we coupled an intrinsic potential habitat modelling approach with a Bayesian network to evaluate the vulnerability of five culturally and economically vital species of Pacific salmon (*Oncorhynchus* spp.) to invasion by northern pike. This study was conducted along 22,875 stream km in the Southcentral region of Alaska, USA. Pink salmon (*O*. *gorbuscha*) were the most vulnerable species, with 15.2% (2,458 km) of their calculated extent identified as “highly” vulnerable, followed closely by chum salmon (*O*. *keta*, 14.8%; 2,557 km) and coho salmon (*O*. *kisutch*, 14.7%; 2,536 km). Moreover, all five Pacific salmon species were highly vulnerable in 1,001 stream km of shared habitat. This simple to implement, adaptable, and cost-effective framework will allow prioritizing habitats for early detection and monitoring of invading northern pike.

## Introduction

Biological invasions are a leading cause of native freshwater biological diversity loss worldwide [1]. Freshwater ecosystems are especially vulnerable to invasions as the human assisted spread of non-native species has transformed freshwater communities at a much higher rate than their terrestrial counterparts [2]. A subset of introduced non-native species have become invasive and have caused substantial environmental and economic impacts [3], with the latter estimated to amount to billions of US dollars per year [4]. The costs of indirect environmental effects caused by these invasions, such as reduction in available prey items or resources, are more difficult to quantify. As the impacts of invaders can vary across broad landscapes, effective management strategies for the conservation of native species and management of non-native species must be useful across large geographic extents [5]. However, management strategies should also allow fine scale site identification for monitoring and suppression, or eradication efforts after introductions are detected.

Fish species are sometimes introduced via intentional methods such as illegal introductions by humans to boost recreational fishing opportunities [6]. Introductions can lead to detrimental consequences for native fauna including competition with other prized sportfish or extirpations of native fishes [7]. Illegal stocking has led to the rapid range expansion of numerous species and resulted in reduced diversity and homogenization of freshwater fauna worldwide [8–10]. Introductions can lead to complex ecological consequences, even in relatively pristine ecosystems, especially when the invader is a top predator [11, 12].

The highly predatory northern pike (*Esox lucius*; hereafter referred to as pike) has been introduced globally [13]. In Alaska, USA, pike are an ecologically and culturally important native fish species north and west of the Alaska Mountain Range, but do not naturally occur south of the range except for a presumed post-glacial relict population near Yakutat, Alaska [13]. In their natural range, pike and Pacific salmon regularly coexist. In the Bristol Bay region (Western Alaska), which hosts the world’s largest sockeye salmon (*O*. *nerka*) runs, pike are numerous. A mechanism to explain this coexistence is spatial segregation through habitat heterogeneity [14]. In the 1950s, a floatplane pilot purportedly translocated pike from a native population in Minto Flats (Yukon River watershed), to Bulchitna Lake in the Susitna River basin. This event is considered the initial source of pike in the Matanuska-Susitna basin [14]. Additional illegal stocking events occurred in the 1960s into Alexander Lake in the Susitna River basin, and to lakes on Alaska’s Kenai Peninsula in the 1970s, resulting in further establishment of pike populations outside of their natural range in Alaska. Despite widespread concern about the impacts of pike, particularly on native Pacific salmon (*Oncorhynchus* spp.), no broad scale assessments of current and future impacts of pike in Southcentral Alaska have been conducted. A regional assessment of the future of the invasion will aid the implementation of effective and proactive detection and monitoring efforts by identification of critically vulnerable areas.

In Southcentral Alaska, the environmental impacts of the pike invasion have led to the rapid decline of multiple salmonid (salmon and trout) species [14] in some locations, and the extirpation of a rare weakly-armored threespine stickleback (*Gasterosteus aculeatus*) ecotype in Prator Lake, Alaska [15]. Extirpation of species by introduced piscivores can result from prey naivety to a novel predator [16] in habitats that lack sufficient shelter from predation. Freshwater fish colonized Southcentral Alaska following the end of the last glacial epoch, thus these communities have existed thousands of years without pike. As a result, they may be especially vulnerable to pike predation and local extirpations are likely to continue as pike spread throughout the invaded Matanuska-Susitna basin.

Pike require slow-moving, shallow, well-vegetated aquatic habitats to complete their life cycle and rely on vegetation for embryo and larval development [17, 18]. This habitat type is common to Southcentral Alaska, where it also plays a key role in the life cycle of juvenile salmon [19]. Juvenile salmon are a preferred prey of pike in invaded areas [20, 21] due to pike’s preference for soft-rayed fishes [22] and their overlapping habitat use with juvenile salmon [23]. Given the extent of this habitat and limited resources available for management, determining the location of available habitat is a crucial first step in predicting future impacts of pike. Further, management decisions can be informed by considering the total amount of habitat available and specific sites within the region that may be impacted by invasion.

The specific goal of this study was to provide the first assessment of the vulnerability of five Pacific salmon species (Chinook, *Oncorhynchus tshawytscha*; chum, *O*. *keta*; coho, *O*. *kisutch*; pink, *O*. *gorbuscha*; and sockeye, *O*. *nerka*) to the ongoing invasion of pike in Southcentral Alaska, with the ultimate aim of guiding conservation prioritization efforts. Across the invaded and potentially invaded range of the Matanuska-Susitna basin, our objectives were to: 1) estimate the location, quantity, and potential of suitable pike habitat, 2) assess the overlap of high-potential juvenile salmon and pike habitat using habitat suitability models, 3) quantify natural and human-mediated colonization potential throughout the stream network, and 4) combine these factors into a Bayesian network to estimate the vulnerability of each salmon species to pike invasion.

## Methods

### Study area

**Fig 1:**
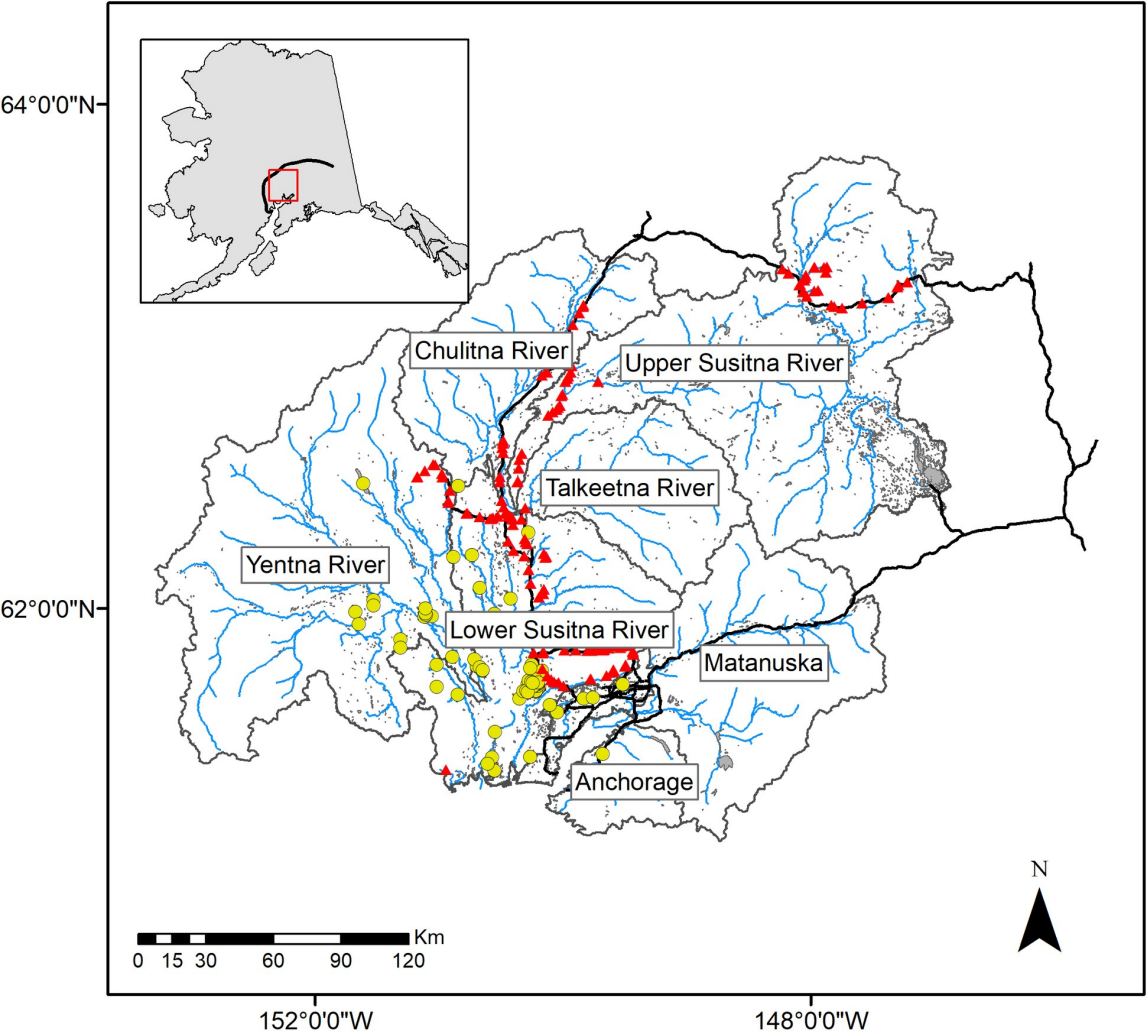
Map of Matanuska-Susitna basin, Southcentral Alaska, USA. Stream reaches are represented as blue lines, sub-basin delineations as grey lines, barriers to fish passage as red triangles, major roads as black lines, significant lakes as grey polygons, and known pike-invaded lakes as yellow circles. The approximate location of the Alaska Range is shown in the inset as a black line.

The Matanuska-Susitna basin covers approximately 63,000 km^2^ in Southcentral Alaska and is composed of two major watersheds, the Matanuska and the Susitna, that drain major portions of the southern Alaska Range mountains (Fig 1). Formed though glacial processes, the basin is flanked by mountain ranges and drains into Cook Inlet to the south. The riverine landscape is predominantly lowlands and contains thousands of lakes and ponds and over 38,000 km of streams and rivers. These complex and mostly intact habitats support a diversity of native fishes. Pike, introduced in the 1950s, are widespread in certain portions of the Matanuska-Susitna basin [24].

### Model of Pacific salmon vulnerability

We used a Bayesian network approach to assess the vulnerability of five Pacific salmon species to invasion by pike in the Matanuska-Susitna basin, Alaska. Bayesian networks provide formal decision support for natural resource issues and can incorporate habitat suitability models [25] and climate scenarios [26] to provide quantitative solutions to ecological problems (reviewed in McCann et al [27]). Bayesian networks allow for the integration of qualitative and quantitative information from various sources to predict outcomes for different scenarios [28]. Moreover, Bayesian networks allow uncertainty to be tracked throughout the network and are easily modified to incorporate new information or data as they become available [29]. As a result, Bayesian networks are useful conservation tools with which to assess the vulnerability of species to ongoing invasions where new information is regularly available and management decisions often require multiple types and sources of data.

**Fig 2:**
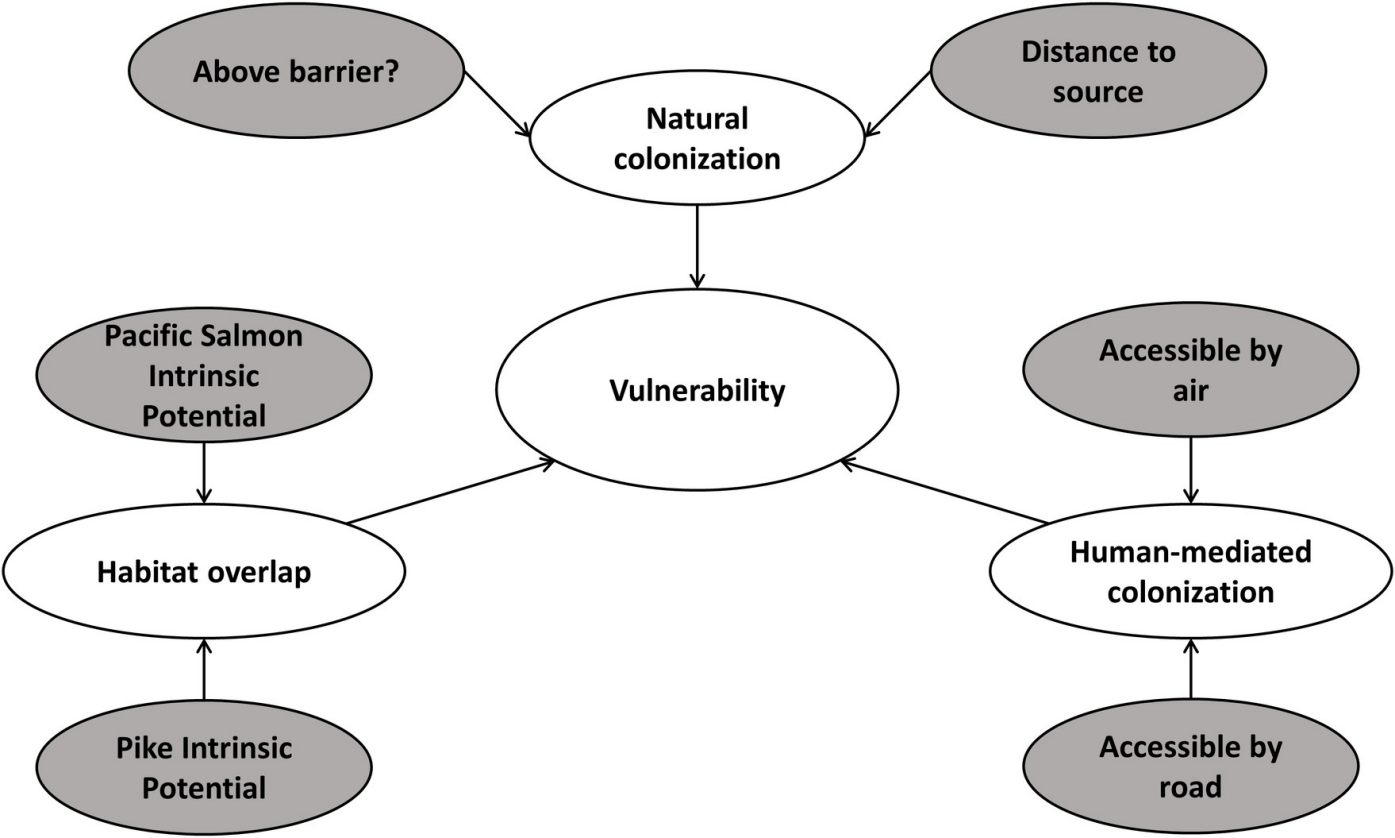
Conceptual diagram depicting factors hypothesized to affect Pacific salmon vulnerability to invasion by northern pike in the Matanuska-Susitna basin (Southcentral Alaska, USA). Shaded ovals represent input variables in the Bayesian network. See Table 1 for definitions of nodes and states within nodes.

Three main variables, or nodes, were included to quantify vulnerability of Pacific salmon to pike invasion using the Bayesian network: natural colonization, human-mediated colonization, and habitat overlap (Fig 2). We created conditional probability tables for each node within the Bayesian network (S1–S3 Tables) to quantify the response and uncertainty from parent nodes to each child node. We populated conditional probability tables from published and unpublished data and expert judgment and used Netica version 6.04 (Norsys Software Corp. 2017) to create and visualize the Bayesian network and conditional probability tables. Node names and states are shown in S4 Table. Input node structure and associated conditional probabilities, except for the Pacific salmon intrinsic habitat potential node, remained constant among salmon species.

Statistical analyses were performed using R version 3.5.0 [30]. Spatial analyses were conducted in R and ArcMap 10.4 (Environmental Systems Research Institute 2015, Redlands, California). Spatial data used and produced by this study are available from the U.S. Geological Survey at https://doi.org/10.5066/P9UJAH16 [31].

### Model components

#### Stream network

Stream attributes were derived from a synthetic stream network created for the Matanuska-Susitna basin (NetMap; [32]). Clarke et al. [33] describe the procedure used to generate NetMap stream attributes using flow accumulation and channel delineation algorithms. The final stream network consisted of ca. 100 m (mean = 98.7 m ± 11.5 m [SD]) stream reaches attributed with geomorphic characteristics such as gradient (%), reach width (m), floodplain width (km), and drainage area (km^2^).

The final Matanuska-Susitna basin stream network extent used in this analysis consisted of 22,875 km of streams with drainage area > 5 km^2^; this was a conservative estimate of the channel initiation threshold representative of the region. The study area contained reaches with a mean gradient of 0.029% (SD = 0.051%) and mean elevation of 556 m (SD = 416 m). The Matanuska-Susitna basin contained 18,719 lakes with a mean area of 0.045 km^2^ (SD = 0.840 km^2^). On average, lakes had a maximum length of 236 m (SD = 420 m) and a fetch of 216 m (SD = 326 m).

#### Habitat overlap

Habitat suitability models based on stream geomorphology, termed intrinsic potential (IP; [34]) models, have been developed to provide estimates of potential habitat for a species. This method allows for characterization of fish habitat quality at the stream reach scale (100–1000 m), over large portions of the landscape. Intrinsic potential models have been used to predict the distribution of salmonid habitat in the Pacific Northwest region of the conterminous U.S. and Alaska [34–36]. Generally, IP models use static, reach-scale, geomorphic attributes to assign a suitability value for a species [34]. Suitability values are generated based on previous knowledge of an organism’s habitat preferences and requirements. Intrinsic potential models can be generated for different life stages [37, 38], allowing for a greater understanding of the impacts of predation on juveniles or sensitivity to habitat alteration for adults [34].

We used the IP approach to estimate habitat potential for pike and five Pacific salmon species across the Matanuska-Susitna basin. Burnett et al. [34] used a two-step process for converting stream reach attributes to IP scores. The first step was to map reach-scale attributes onto a zero to one scale using suitability curves, where zero represents unsuitable and one represents fully suitable. Burnett et al. [34] recommended using three attributes as optimal for IP calculations. Next, the overall IP score is calculated as the geometric mean of the reach-specific suitability rankings for each of the selected attributes (Eq 1).


$$IP_{x}=\sqrt[{3}]{IP_{1x}*IP_{2x}*IP_{3x}}$$ (1)

Thus, the least suitable attribute carried the most overall weight in determining habitat suitability and zero values for any attribute resulted in an overall IP equal to zero (i.e., low or no habitat potential). In the following section, we provide a detailed description of how IP was calculated for Pacific salmon and pike in the Matanuska-Susitna basin. For all IP model development, we relied on previously identified fish-attribute relationships or expert judgment when empirical data were not available.

**Fig 3:**
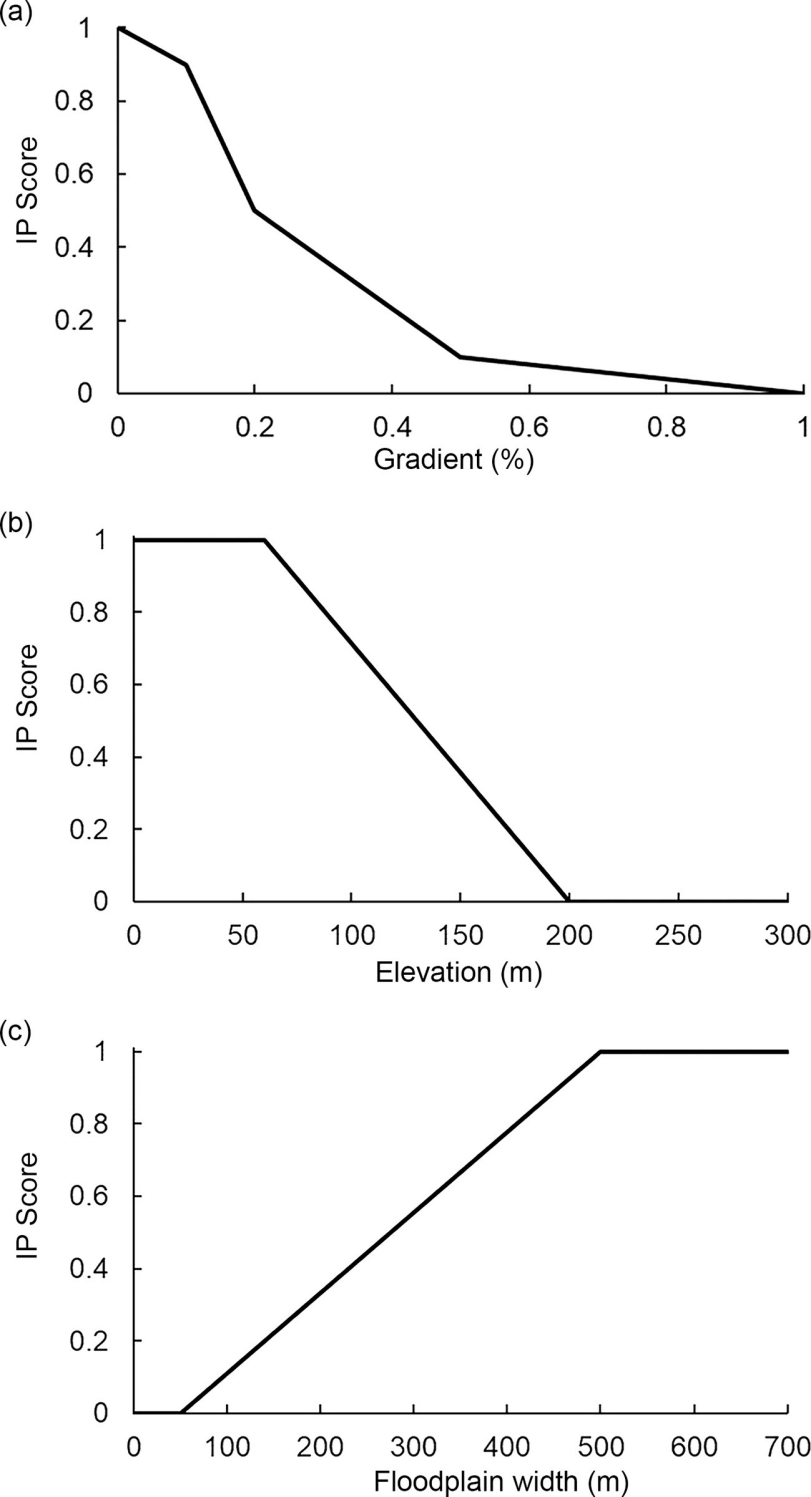
Intrinsic Potential (IP) curves for northern pike for three attributes. (a) gradient (%); (b) elevation (m); and (c) floodplain width (m).

We created an IP model for invasive pike in the Matanuska-Susitna basin based on a combination of expert judgment and by examining relationships between pike occurrence and geomorphic attributes from within their native range. Inskip [39] hypothesized that pike should most commonly occur in low-gradient stream reaches due to the species preference for low-velocity, shallow waters, with little or no suitability in areas with gradient greater than 0.5% (Fig 3A). Second, elevation is a predictor of pike in their native range in Alaska [40]. Elsewhere, it is commonly used to predict fish distributions [41–43]. Elevation may serve as a proxy for climatic and physicochemical attributes (e.g., temperature, water chemistry; [44, 45]) or position along the stream continuum [43]. The index curve for elevation assumed habitat suitability to be zero above 200 m elevation (Fig 3B). Although it is possible for invasive pike to occur above the 200 m elevation threshold as a result of human-assisted translocation to high-elevation sites, such events are unlikely to generate established pike populations owing to likely unsuitable environmental conditions in the area. Finally, floodplains provide access and connectivity to the complex, well-vegetated habitat types that are crucial for pike spawning and juvenile rearing [17, 18, 46]. Indirect measurements of floodplain accessibility have been incorporated in pike habitat suitability models (e.g., percent pools and backwaters during summer, [39]; wetland type, [44]; and percent of lakes in the watershed, [40]) but the model presented here is the first to utilize reach-scale measurements. Specifically, we created an index curve where pike habitat suitabilitiy increased with floodplain width (>500 m; Fig 3C). We used these three curves to calculate final pike IP scores for stream reaches in the Matanuska-Susitna basin using the NetMap tools extension for ArcGIS [32].

We applied existing juvenile Pacific salmon rearing habitat IP models to the Matanuska-Susitna basin study area to assess potential for overlap between habitat areas used by rearing salmon and pike. Here, salmon rearing habitat is defined as the freshwater habitat in which juvenile salmon have adequate food and shelter to survive and grow. Habitat suitability models for sockeye and coho salmon [47] were modified for compatibility with the NetMap synthetic stream network and to more closely follow the IP methodology. Specifically, references to barriers to fish passage and known species distribution data were removed from calculations. Remaining attributes included mean annual flow (m^3^∙s^-1^), gradient (m∙m^-1^), and glacial area (% coverage). The IP models developed for pink and chum salmon in Southeast Alaska included gradient (m∙m^-1^), mean annual flow (m^3^∙s^-1^), and valley-width index [48]. Finally, an IP model for Chinook salmon in the Copper River watershed (Southcentral Alaska; [35]), was applied to the Matanuska-Susitna basin. Attributes included in the model were mean annual flow (m^3^∙s^-1^), gradient (m∙m^-1^), and glacial area (% coverage).

We assessed the probability of habitat overlap between pike and the five salmon species by classifying habitat IP into three categories (low, moderate, high). Intrinsic potential for pike and Pacific salmon were classified based on a 0–1 scale (low = 0–0.25; moderate = 0.25–0.75; high = 0.75–1.0). We based the conditional probability table for habitat overlap on known relationships between salmon and pike, and expert judgment (S1 Table).

#### Natural colonization

Since the first introductions, pike have dispersed and colonized throughout the Susitna stream network and are currently established in at least 75 lakes and numerous waterways in Southcentral Alaska [49]. Future colonization of new waterbodies by pike was modeled as a function of the species’ estimated dispersal abilities and tempered by known barriers to movement. We used the Alaska Department of Fish and Game (ADFG) Northern Pike Waters Catalog [49] to identify all lakes with known pike populations (status = Known, or Suppression) that were connected to the stream network (Connectivity = open outlet, intermittent outlet, barriered outlet, or flood prone). Invaded lakes were considered putative source populations and the hydrologic distance (km) from the closest source population to each stream reach was calculated using STARS version 2.0.6 [50] toolbox in ArcMap (S1 File).

The *fishmove* (0.3–3) R package [51] was used to fit a pike-specific leptokurtic dispersal kernel [52] and estimate the probability of pike occurrence as a function of distance from source. Predictions were based on parameters from a multiple regression of four variables [51]: fish length (mm), caudal fin aspect ratio, stream order [53], and time (days). We set the average fork length to 450 mm which approximates the overall average length in many populations in the Matanuska-Susitna basin [54], time was set to 365 days, and caudal fin aspect ratio and stream order were calculated using default values for pike (1.39 and 6^th^, respectively; [55]).

The ADFG Fish Passage Inventory Database (FPID) was used to identify probable anthropogenic barriers to natural pike movement in the Matanuska-Susitna basin [56]. The FPID ranks culverts on their ability to allow passage of juvenile coho salmon (see [56] for detail), which we assumed was relevant for pike since the species are weak swimmers [57]. Devil’s Canyon (62.826417, -149.36673) is a known velocity barrier to most anadromous fishes, thus was considered a barrier to the upstream migration of pike.

We used the leptokurtic dispersal kernel established using *fishmove* and examined the location of known barriers to fish movement to calculate the probability of natural colonization. We grouped in-stream distances to the closest known pike source into three categories (close: < 1,000 m; moderate: 1,000–10,000 m; far: > 10,000 m), based upon pike movement capability. We classified stream reaches into two groups: above (1) or below (0) known barriers to fish passage. The natural colonization node consisted of four states (none, low, moderate, and high; S2 Table).

#### Human-mediated colonization

Two major anthropogenic vectors implicated for pike dispersal throughout the Matanuska-Susitna basin are movement by air and road [14]. Because road access to waterbodies is limited in the region, and single-engine aircraft have been identified as a dispersal mechanism for invasive plants [58] and the original vector for pike in the region, it stands to reason that the potential for pike introduction by air continues to exist. We used data from [58] to identify lakes accessible by single-engine aircraft and thus susceptible to pike introduction by air.

We calculated lake fetch for 18,719 lakes within the Matanuska-Susitna basin as it is a key variable that determines a pilot’s ability to safely land on a given lake (S2 File). Float plane-accessible lakes were ranked from one to five according to the type of aircraft supported; for example, lakes of rank two support two classes of aircraft. The Matanuska-Susitna basin dataset was divided into unique 12-digit hydrologic unit codes (HUC; hereafter ‘subwatershed’; [59] and we summed the ranks of all lakes within each subwatershed. Lakes residing along the boundary of multiple subwatersheds were counted towards the total for each. Finally, we assigned the summed lake rank value to all stream reaches within the bounds of each subwatershed.

Anthropogenic infrastructure such as boat launches or public waterbody access sites are commonly used to predict presence of invasive aquatic species [5, 60, 61]. Limited data are available regarding illegal stocking of waterbodies as a function of distance to trails, which are common throughout the study area. However, waterbodies close to roads are more likely to contain invasive species as a result of human-mediated introductions relative to waterbodies located farther away [62–64]. We used Euclidean distance from an individual stream reach (m) to the nearest road as a proxy because data on boat launches and access sites were not available for the study area. We identified 4,776 km of major roadways and trails, all of which serve as possible conduits for pike introductions [65].

To assign input node probabilities for overall human-mediated colonization, we binned measurements of distance from each reach to the closest road into three categories, close (< 1,200 m), moderate (1,201–3,600 m), and far (>3,600 m; Table 1) and grouped the sum of plane-accessible lakes into four categories (none: 0; low: 0–10; moderate: 11–20; high: > 20). The human-mediated colonization node (Fig 2) consisted of three states (low, moderate, and high; Table 1, S3 Table).

**Table 1:** Node definitions and states for a Bayesian belief network (Fig 2) to assess vulnerability of juvenile salmon to introduced northern pike in the Matanuska-Susitna River basin, Southcentral Alaska, USA.

Node Name	Definition	State
Natural colonization	Potential for northern pike to colonize by natural means	none
		low
		moderate
		high
Distance to invaded waterbody (I)	In-stream distance to nearest invaded lake	close: < 1,000 m
		moderate: 1,000–10,000 m
		far: > 10,000 m
Above barrier? (I)	Whether the stream reach is located above a known barrier	yes: 1
		no: 0
Human-mediated colonization	Potential for northern pike to be introduced by humans	low
		moderate
		high
Accessible by road (I)	Potential for introduction by roadway	close: < 1,200 m
		moderate: 1,200–3,600 m
		far: > 3,600 m
Accessible by plane (I)	Potential for introduction by five types of single-engine aircraft, common to the Matanuska-Susitna basin as measured by the sum of lake ranks within a HUC-12 unit	none: 0
		low: 0–10
		moderate: 11–20
		high: > 20
Habitat overlap	Potential for overlap of different quality habitat between northern pike and salmon	low
		moderate
		high
Intrinsic potential northern pike	Habitat potential for northern pike as measured for a given reach using gradient, elevation, and floodplain	low: < 0.25
		moderate: 0.25–0.75
		high: > 0.75
Intrinsic potential Pacific salmon (I)	Habitat potential for Pacific salmon as measured for a given reach	low: < 0.25
		moderate: 0.25–0.75
		high: > 0.75
Vulnerability to invasion	Vulnerability to invasion by northern pike, for each Pacific salmon	low
		moderate
		high

Note: Input nodes (I) are assigned the probability of being in each state.

#### Species-specific vulnerability

Overall vulnerability to invasion by pike was calculated separately for each Pacific salmon species as a function of the three major nodes (habitat overlap, natural colonization, and human-mediated colonization) and their input nodes. The output probability was classified into three states (low, moderate, or high) based on a weighted conditional probability table (S4 Table). We built the conditional probability table by assigning weights to the classes within the three major nodes, weighting both within and among nodes (S5 Table). We assumed habitat overlap to be the most important factor followed by natural colonization, and human-mediated colonization. Finally, we projected the states from each major node onto the river network by exporting the terminal node and summed the total length (km) of stream reaches falling into each category (low, moderate, high) within each of the nodes: habitat overlap, human-mediated colonization, natural colonization, and vulnerability. We estimated vulnerability uncertainty for each reach as the standard deviation of the expected value predicted by the Bayesian network.

#### Model sensitivity

We performed a sensitivity analysis within Netica to determine the influence of input variables on each outcome variable in the model. The degree of sensitivity of one node to another was calculated using the mutual information (i.e., entropy reduction) method.

## Results

### Habitat potential

**Fig 4:**
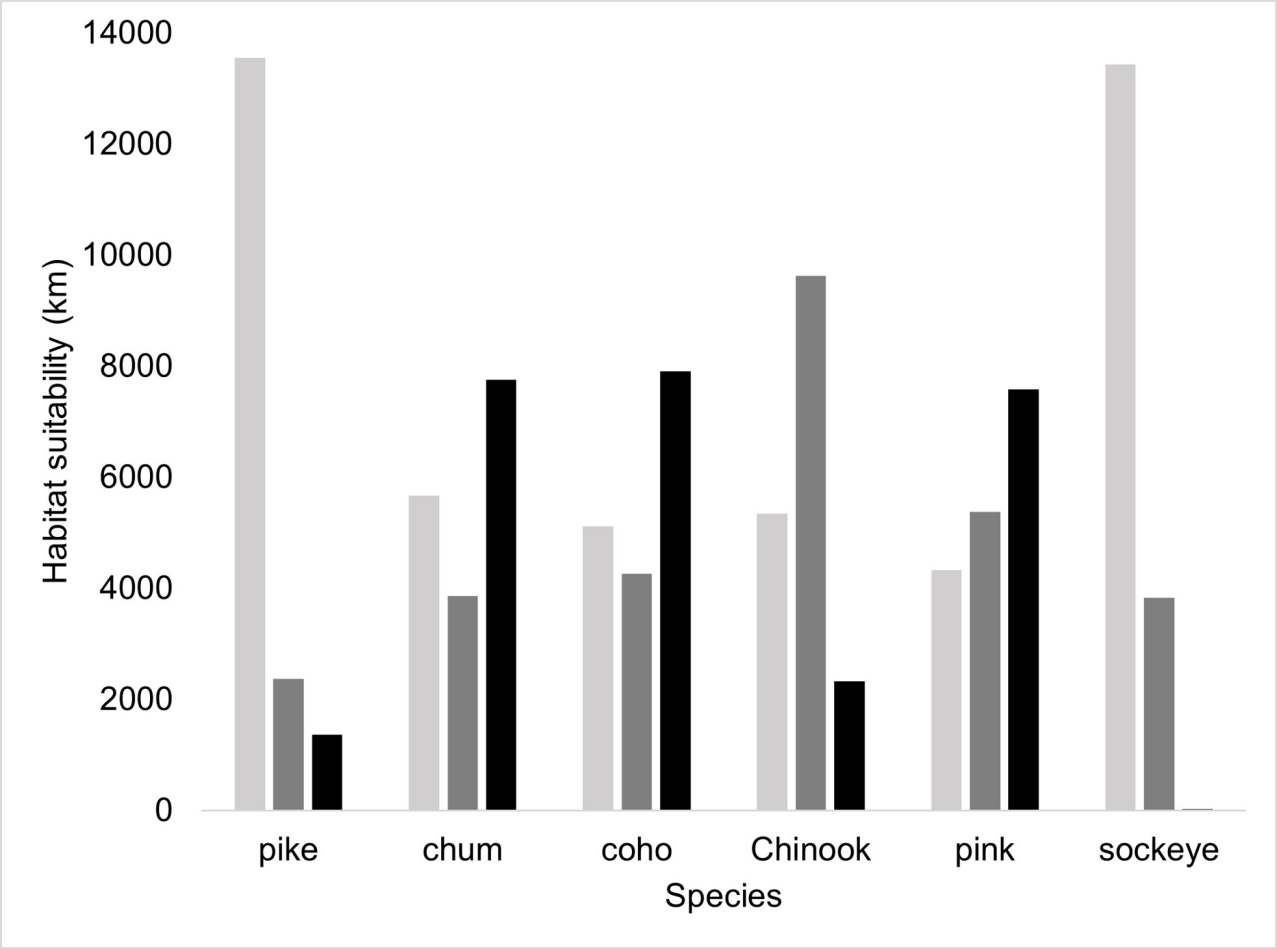
Sum of stream reach habitat suitability (km) for northern pike and Pacific salmon in the Matanuska-Susitna basin, Alaska, USA. Light grey represents low-potential, dark grey represents moderate-potential, and black represents high-potential habitat.

We estimated 6% (1,364 km) of stream reaches within the Matanuska-Susitna basin to be highly suitable for pike; 84% (1,146 km) were located within the Yentna River and Lower Susitna River subwatersheds (Fig 1). An additional 10% (1,858 km) of the basin was classified as moderately suitable habitat; 78% within the Yentna River and Lower Susitna River subwatersheds. The remainder of the basin was predicted to have low habitat suitability for pike because reaches were at higher elevations (mean = 655 m ± 387 m [SD]) and gradients (mean = 0.035% ± 0.055% [SD]) or had little floodplain width (mean = 197 m ± 378 m [SD]) (Fig 4). All available (n = 94) georeferenced observations of invasive pike (ADFG, unpublished data) fell within reaches predicted to be highly suitable for pike.

Consistent with distinct life history and habitat requirements, Pacific salmon IP scores differed among species (Fig 4). Coho salmon was predicted to have access to the most habitat with high IP (7,904 km), followed closely by chum salmon (7,760 km) and pink salmon (7,585 km). Finally, we predicted 2,326 km of habitat with high IP for Chinook salmon but only 22 km of high IP sockeye salmon habitat due to their reliance on lakes for rearing. Although there was little habitat with high IP for Chinook salmon, this species had the most habitat with moderate IP (9,623 km), followed by pink salmon (5,379 km), coho salmon (4,267 km), chum salmon (3,857 km), and sockeye salmon (3,828 km).

Overlap of habitat with high IP for pike and high IP for rearing salmon differed by species. Coho salmon had the largest overlap (3,555 km of stream reaches; 21% of available habitat) followed by chum salmon (3,450 km, 20%), pink salmon (3,085 km, 18%), Chinook salmon (1,980 km, 11%) and sockeye salmon (1,364 km, 8%). Overlap of habitat with high IP for pike and moderate IP for salmon was more consistent between species. Chinook salmon had the largest overlap (1,237 km; 7%) followed by coho, chum, sockeye, and pink salmon (between 1%– 4%, respectively). For all salmon species, most IP was classified in the “low” probability of overlap class (78% - 89%). There was little predicted habitat overlap upstream of barriers. Chum salmon, coho salmon, and pink salmon each had 107 km of “high” and “moderate” class habitat overlap. There were 95 km of Chinook salmon streams in these two classes and only 24 km of sockeye salmon streams.

### Natural colonization

We identified 67 lakes that may serve as sources of pike colonists within the study area, one natural barrier (Devil’s Canyon, Susitna, AK), and 137 artificial culvert barriers to fish passage. Of the stream reaches excluded by barriers, 200 km were identified as close (<1 km; natural colonization node class = high) to a known pike source but were not likely to be naturally invaded. The leptokurtic dispersal kernel predicted the probability of dispersing 1 km was 16% and 10 km was 4.5%, over a 365-d period.

Approximately 5,586 km (24%) of the Matanuska-Susitna basin was identified as unavailable for natural colonization due to barriers. Pink salmon were the most range-restricted with 2,792 km (37%) of high IP habitat located upstream of known barriers, followed by coho salmon (2,515 km, 32%), chum salmon (2,326 km, 30%), and Chinook salmon (305 km; 13%). However, we identified 4,058 km of moderate IP Chinook salmon rearing habitat above barriers. Coho salmon, pink salmon, chum salmon, and sockeye salmon had much less moderate IP habitat above barriers (range: 771 km– 1,740 km).

### Human-mediated colonization

We found 2,334 km of streams were located within 1,200 m of major Matanuska-Susitna Borough roads (node class = close) and 1,806 km of streams in the moderate node class (1,200–3,600 m). The remaining 19,035 km were located farther than 3,601 m from the nearest road (node class = far). We identified 2,567 lakes that met minimum fetch criteria. Of these, only 266 lakes were excluded based on aircraft range for the smallest aircraft type (rank = 1). Lakes large enough to support aircraft averaged 0.266 km^2^ (SD = 2.26 km^2^) in area, 810 m (SD = 922 m) in length, and average fetch was 623 m (SD = 717 m).

### Species-specific vulnerability

**Fig 5:**
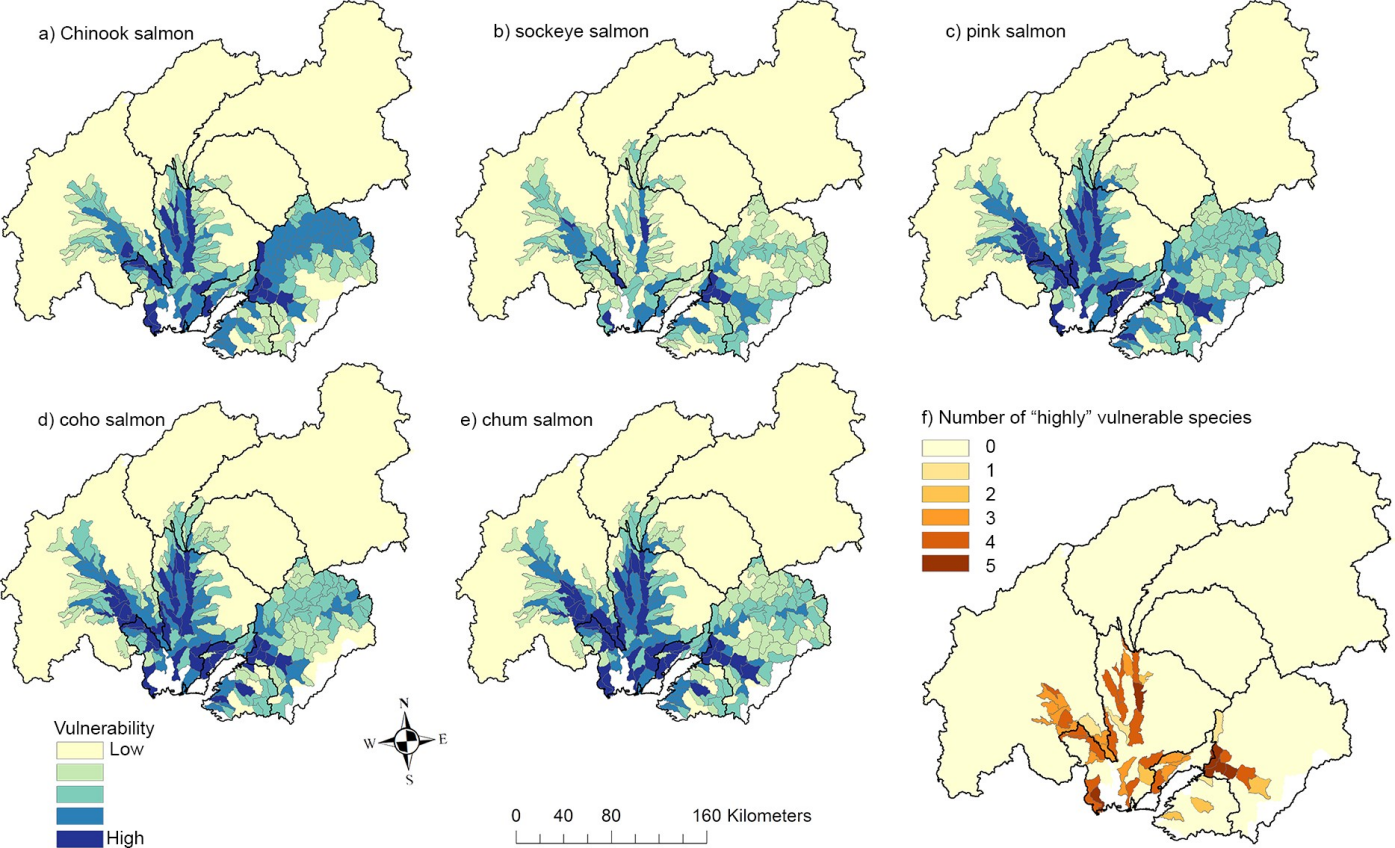
Vulnerability of Pacific salmon to invasion by northern pike for the Matanuska-Susitna basin (Southcentral, Alaska, USA). Species-specific estimates shown in panels a–e and a composite “highly-vulnerable” estimate shown in panel f. Black lines represent sub-basins. Darker colors represent higher vulnerability with species-specific vulnerability shown in blues and the number of species identified as “high” vulnerability shown in oranges.

Chum salmon had the highest risk to invasion, as measured by total stream kilometers in the high vulnerability class (2,557 km), followed by coho salmon (2,534 km), pink salmon (2,458 km), Chinook salmon (1,661 km), and sockeye salmon (1,196 km). Most of the highly vulnerable stream reaches for Pacific salmon were in the Yentna River and Lower Susitna River subwatersheds (78%; Fig 5, Table 2). There were also 12,654 km of streams predicted to have moderate vulnerability to invasion. Chinook salmon had the greatest moderate vulnerability (3,235 km; S6 Table), followed by chum salmon (2,557 km), coho salmon (2,534 km), pink salmon (2,458 km), and sockeye salmon (1,196 km).

**Table 2:** Total length (km) of highly vulnerable stream reaches for five Pacific salmon species to northern pike within the Matanuska-Susitna basin, Alaska, USA by HUC-8 sub-basin.

Sub-basin	Area	Stream length					
(HUC-8)	(km^2^)	(km)	chum	coho	Chinook	pink	sockeye
Anchorage	3061	939	233 (0.09)	250 (0.1)	129 (0.08)	229 (0.09)	116 (0.1)
Matanuska	8662	2393	258 (0.1)	240 (0.09)	147 (0.09)	272 (0.11)	145 (0.12)
Upper Susitna River	16346	5546	13 (0.01)	14 (0.01)	4 (0)	4 (0)	4 (0)
Chulitna River	6728	2280	27 (0.01)	28 (0.01)	11 (0.01)	19 (0.01)	11 (0.01)
Talkeetna River	5286	1681	41 (0.02)	32 (0.01)	13 (0.01)	29 (0.01)	13 (0.01)
Yentna River	15869	5988	557 (0.22)	516 (0.2)	280 (0.17)	548 (0.22)	325 (0.27)
Lower Susitna River	8855	4049	1428 (0.56)	1456 (0.57)	1078 (0.65)	1356 (0.55)	581 (0.49)

Total area (km^2^) and length of streams (km) in each sub-basin are also shown. Values in parenthesis represent the species-specific proportion of highly vulnerable habitat.

Uncertainty surrounding invasion vulnerability ranged from 0.28 to 0.84 (mean = 0.67 ± 0.07 [SD]). Sockeye salmon showed the least uncertainty in vulnerability classification (mean = 0.63 ± 0.05 [SD]), followed by Chinook salmon (mean = 0.66 ± 0.06 [SD]). The mean uncertainty for chum salmon, coho salmon, and pink salmon was approximately 0.69 (± 0.06 [SD]). For all salmon species, reaches with the highest uncertainty were concentrated low in the basin (i.e., Yentna River and Lower Susitna River subwatersheds), and in the Matanuska sub-basin.

Overall, the Bayesian network identified pink, chum, and coho salmon to have similar extents ranked as highly vulnerable (15.2%, 14.8%, and 14.7%, respectively; Fig 5C–5E). Chinook salmon had 10.8% of their predicted extent ranked as highly vulnerable (Fig 5A). Sockeye salmon showed the lowest vulnerability with 8.2% predicted to be highly vulnerable (Fig 5B). Finally, we identified 1,001 km of streams occupied by all five species of Pacific salmon predicted to be highly vulnerable to pike invasion (Fig 5F).

### Sensitivity analysis

A network sensitivity analysis showed that the Bayesian network performed as expected. In the Bayesian network, salmon vulnerability to invasion was most sensitive to habitat overlap potential (variance reduction = 0.1267) and was relatively insensitive to the natural colonization (0.0221) and human-mediated colonization nodes (0.0023).

## Discussion

Managing the impacts of ongoing invasions is appropriately likened to triage medicine, where the need for interventions far exceeds available resources [66]. Here we inform the spatial management of the northern pike invasion of Southcentral Alaska through a flexible modelling approach which is easily extended to other species. Specifically, we combined habitat suitability modelling, estimates of connectivity and human transport that drive species introductions, and Bayesian networks to assess the vulnerability of five Pacific salmon species confronted with a novel top predator. Our approach has broad application both inside and outside of Alaska as pike have been introduced and deemed ‘invasive’ throughout much of their non-native distribution and are currently threatening the persistence of a variety of native fishes (Western United States: [67], Canada: [68], Spain: [69], and elsewhere in the Mediterranean: [70]). In the face of increasing invasions in freshwaters, assessments such as ours provide managers with quantitative methods to assess the impacts of species introductions quickly and efficiently over large geographic areas.

### Intrinsic potential

Intrinsic potential models proved useful to identify IP for northern pike and rearing juvenile salmon over ca. 100 m stream reaches throughout the 63,000 km^2^ Matanuska-Susitna basin. For all IP models, we chose a 0.75 threshold for “high” but recognize that this is subjective and could lead to underestimates of IP [36]. However, we argue that narrow ranges for the “low” (0–0.25) and “high” (0.75–1.0) classes captured true IP, while the range of the “moderate” class (0.25–0.75) represented the uncertainty in fish-geomorphic attribute associations. Further, since all observations of invasive pike occurred within the high IP class, which also corresponded to areas with known impacts on salmonids, there is corroborative evidence that the curves performed adequately to predict potential habitat. Given the decline of Chinook, coho, and sockeye salmon in Southcentral Alaska we recognize and stress the importance of including areas in the moderate IP class in future management actions.

#### Northern pike

Our study indicated a substantial amount of habitat suitable for pike that is unoccupied within the Matanuska-Susitna basin (at minimum 1,000 stream-km), consistent with the pattern of an ongoing invasion. We constructed IP models with three attributes which are important predictors of pike throughout their native range (Fig 3). Our approach assumes that invasive pike have similar habitat requirements as their native counterparts. This assumption is reasonable as native and invasive pike are found in similar habitats and the association between habitat and pike is well documented throughout their range.

This is the first construction of an IP model for pike. Because the model was constructed for an area with limited pike distribution data it was not possible to statistically verify the suitability curves for our study area. Future pike monitoring and distribution assessments could add information to refine the pike IP model.

#### Pacific salmon

Our assessment of Pacific salmon rearing habitat demonstrated a large quantity of potential habitat for all species and generally aligned with previously conducted suitability estimates for the region [47]. The pink salmon and chum salmon IP models were parameterized for streams in Southeast Alaska [48] and care should be taken when applying IP models from outside the study area. That caveat notwithstanding, recent implementations of IP models for salmon species suggest that species- or life stage-specific IP models are robust outside the area for which they were parameterized [36]. Our analyses would benefit from further work to ground-truth pink and chum salmon distribution in Southcentral Alaska.

We are unaware of sockeye salmon IP models due to their complex life cycle and reliance on lakes for rearing. Sockeye salmon in the Matanuska-Susitna basin can be exposed to heavy predation during the rearing period if pike are also present [71] and are highly vulnerable as smolts during seaward migrations if pike habitat is located downstream (i.e., near the outlet) of rearing lakes. Due to a limited IP model for sockeye salmon, the vulnerability estimates we produced are likely an underestimate of the vulnerability of this species to pike. As such, further development of a lake-rearing sockeye salmon IP model would be informative.

### Vulnerability assessment

Our vulnerability assessment provides a framework to identify hotspots along the stream network, at an appropriate spatial scale, where fisheries managers can focus monitoring or eradication efforts. We identified critically vulnerable areas, shared by multiple salmon species, by calculating vulnerability across the entire stream network at a relatively fine spatial scale (ca. 100 m reach), which can easily be scaled up (i.e., averaged, or aggregated) to the watershed, sub-basin, or basin levels. This form of triage is crucial for managing invasions across large geographic scales as it provides the information necessary to design effective suppression strategies that maximize impacts given limited resources. This is particularly crucial in areas like Southcentral Alaska where access by road is limited thus costs associated with field work increase dramatically.

In this vulnerability assessment, estimates of human-mediated colonization by air were limited to lakes within the flight range of common aircraft types in the study area. We acknowledge that smaller aircraft could originate from lakes throughout the Matanuska-Susitna basin but only one class of aircraft was limited by range from the calculated flight origin (Lake Hood, Anchorage, Alaska). Similarly, we acknowledge that distance to roads and increased propagule pressure could be confounded by other forms of disturbance (e.g., urbanization near lakes) that increase the likelihood of illegal introduction and were not evaluated in this analysis.

Much uncertainty surrounds the dispersal ability of pike. As Skov et al. [72] reviewed, some studies indicate that pike is a sedentary species, dispersing only meters each day, and yet other work has shown pike capable of dispersing up to 26 km d^-1^. Estimates of natural colonization were verified using radio telemetry data from pike in their native range (Yukon River, AK; ADFG, unpublished data). These telemetry data showed that pike exhibit patterns of movement like many other stream fish species, with some of the population remaining sedentary and a few individuals demonstrating long-distance dispersal [73]. A possible bias in this dispersal kernel is the reliance on individuals sufficiently large enough for radio tagging; it is unknown if smaller or young individuals disperse at different rates. Although we limited dispersal to 365 days, results from extended dispersal kernels suggested that pike will continue to colonize Southcentral Alaska given time. We note that our movement estimates were conservative because we modeled mean and not maximum distances.

We limited our analyses to stream reaches located below barriers to fish passage and Devil’s Canyon on the Susitna River. However, little is known regarding pike jumping ability, so the ability to bypass barriers is unknown. It is noteworthy that while introduction above barriers is possible, the pike IP model predicted only 106 km of moderate or high IP in reaches upstream of barriers. Thus, the risk of further pike invasion due to culvert bypass is relatively small when compared to total available habitat.

### Study limitations and uncertainties

Generally, uncertainty estimates from the Bayesian network were higher in the Yentna River, Lower Susitna River, and Matanuska River sub-basins relative to the other sub-basins. As the natural and human-assisted colonization node states were similar among species, changes in uncertainty here represent changes in IP estimates for each species and their overlap with pike. Narrowing the range of the “moderate” IP class (i.e., IP = 0.25–0.75) may reduce uncertainty associated with the predictions. Also, future work to validate and refine the pike and salmon IP models could allow for a more accurate representation of true habitat potential in the Matanuska-Susitna basin, which in turn would reduce the uncertainty in the vulnerability estimates.

This vulnerability assessment does not directly consider life history differences among salmon species, particularly differences in the length of the freshwater rearing period during which anadromous salmon may be exposed to pike predation. Given this, pink salmon and chum salmon, which we identified as highly vulnerable based on habitat overlap, may be less affected because they migrate directly to sea upon emergence [74]. Thus, depending on species life-history characteristics, temporal overlap may be minimal, and vulnerability may be less than predicted. Pike have been documented to prey on juvenile salmon in brackish waters [75], and have been captured in nets in Cook Inlet downstream of our study area (T. Shilling, Northern District Set Netter of Cook Inlet, personal communication), thus the potential for predation on juvenile salmon outside of freshwater in Southcentral Alaska exists. However, it seems logical that rearing is the most vulnerable salmon life-stage because the greatest predation occurs on juveniles during their freshwater residency, though information of the specific critical stages or bottlenecks of salmonids by pike are not well known.

Finally, the salmon IP models are specific to juvenile rearing and do not consider out-migration, or the period in which juvenile salmon travel from freshwater to the ocean. This period of downstream movement could expose juveniles to predation. The extent of predation is dependent on time spent in proximity to a predator, the number of predators present, and prey density [76]. Hence, a fine-scale understanding of the relative spatial locations and movement patterns of predator and prey are crucial to accurately evaluate the extent of predator-prey interactions. IP model predictions occur over spatial scales (ca. 100 m reach) that do not allow for examination of such fine-scale patterns. For example, in the Matanuska-Susitna basin, as the prey (juvenile salmon) move downstream and into the mainstems of larger rivers, the predators (pike) are likely constrained to slower moving, off-channel habitats within those rivers. So, while a stream reach may exhibit high pike and salmon IP, the two species may not overlap in space due to different microhabitat utilization. Further, many large rivers in the Matanuska-Susitna basin are glacial and highly turbid so visual predators such as pike [77] are likely at a disadvantage during the spring months when salmon smolt out-migrate and river flows are high and turbid. We expect that out-migrating salmon might find refuge and reduce risk of predation by moving into, traveling through, and rearing in habitat unsuitable for pike. Future work to investigate the frequency and duration at which pike move into sub-optimal habitats to pursue out-migrating smolt is warranted.

### Management implications

Persistence and sustainability of Pacific salmon are vital to the preservation of economies, ecosystems, and cultures in Alaska. Low runs of salmon in Southcentral Alaska have led to emergency orders and pre-emptive closures of sport and commercial Chinook salmon fisheries by ADFG during recent fishing seasons [78]. Examining the habitat overlap between invasive pike and salmon populations is a critical step to help inform proactive management to the invasion with the goal of mitigating the current and future impacts of non-native northern pike.

Although the invasion in the Matanuska-Susitna basin is already widespread, we identified additional uncolonized reaches with highly suitable pike habitat. Therefore, it seems likely that the invasion will continue to expand. Recent ADFG management efforts have been successful at suppressing or eliminating pike from closed lakes and several streams, but suppression is a lengthy and costly process that must be continued indefinitely. Moreover, managers must continually monitor the system for new invasions via natural and human-mediated colonization [14]. For example, Spens et al. [79] found that given opportunity (i.e., time) pike colonized lakes in Sweden regardless of distance from source. Because Southcentral Alaska was recently colonized, identifying suitable pike habitat and habitat in which multiple salmon species are vulnerable will prove crucial in developing management plans to respond to the invasion. Finally, although we know that pike are not the sole or, in many cases, primary threat to Pacific salmon populations in Southcentral Alaska, our results demonstrate a clear overlap between pike and salmon in this region. Specifically, as pike continue to invade suitable habitat the species is likely to interact with naïve salmon and may further impact salmon production.

## Supporting information

S1 Table Conditional probability table for habitat overlap of Pacific salmon with northern pike in the Matanuska-Susitna basin, Alaska, USA. (DOCX)

S2 Table Conditional probability table for natural colonization of northern pike in the Matanuska-Susitna basin, Alaska, USA. (DOCX)

S3 Table Conditional probability table for human-mediated colonization of northern pike in the Matanuska-Susitna basin, Alaska, USA. (DOCX)

S4 Table Conditional probability table for vulnerability of Pacific salmon in the Matanuska-Susitna basin, Alaska, USA. (DOCX)

S5 Table Weighting for conditional probability table for vulnerability of Pacific salmon in the Matanuska-Susitna basin, Alaska, USA (S4 Table). Ranks were weighted as follows: habitat overlap (Input node = habitat; high = 100), natural colonization (Input node = natural; high = 50), and human-mediated colonization (Input node = human; high = 10). The total possible weighted rank was 160, thus we used 80 as the inflection point for shifting vulnerability from low towards high.
(DOCX)

S6 Table Vulnerability of Pacific salmon by HUC-8 sub-basin in stream kilometers within the Matanuska-Susitna basin, Alaska, USA. Table represents Netica vulnerability class ‘moderate’.
(DOCX)

S1 File Creation of the landscape network. (DOCX)

S2 File Calculation of lake fetch. (DOCX)
