# Supplementary material for: Vulnerability of Pacific salmon to invasion of northern pike (Esox lucius) in Southcentral Alaska
Source: PLoS One. 2021 Jul 2;16(7):e0254097. doi: 10.1371/journal.pone.0254097 (PMC8253411; doi:10.1371/journal.pone.0254097)
Supplement: S1 Table — (DOCX) [file pone.0254097.s001.docx]

**S1 Table. Conditional probability table for habitat overlap of Pacific salmon with northern pike in the Matanuska-Susitna basin, Alaska, USA.**

| **Input node** | | **State (Habitat overlap)** | | |
| --- | --- | --- | --- | --- |
| **IP_PIKE** | **IP_SALMON** | **low** | **moderate** | **high** |
| low | low | 100 | 0 | 0 |
| low | moderate | 60 | 40 | 0 |
| low | high | 50 | 30 | 20 |
| moderate | low | 60 | 40 | 0 |
| moderate | moderate | 0 | 100 | 0 |
| moderate | high | 0 | 30 | 70 |
| high | low | 0 | 25 | 75 |
| high | moderate | 0 | 15 | 85 |
| high | high | 0 | 0 | 100 |
