# Supplementary material for: Vulnerability of Pacific salmon to invasion of northern pike (Esox lucius) in Southcentral Alaska
Source: PLoS One. 2021 Jul 2;16(7):e0254097. doi: 10.1371/journal.pone.0254097 (PMC8253411; doi:10.1371/journal.pone.0254097)
Supplement: S2 Table — (DOCX) [file pone.0254097.s002.docx]

**S2 Table. Conditional probability table for natural colonization of northern pike in the Matanuska-Susitna basin, Alaska, USA.**

| **Input node** | | **State (Natural colonization)** | | | |
| --- | --- | --- | --- | --- | --- |
| **BARR** | **DISTPIKE** | **none** | **low** | **moderate** | **high** |
| yes | close | 10 | 50 | 35 | 5 |
| yes | moderate | 10 | 70 | 15 | 5 |
| yes | far | 15 | 80 | 5 | 0 |
| no | close | 0 | 0 | 0 | 100 |
| no | moderate | 0 | 0 | 100 | 0 |
| no | far | 0 | 100 | 0 | 0 |
