# Supplementary material for: Vulnerability of Pacific salmon to invasion of northern pike (Esox lucius) in Southcentral Alaska
Source: PLoS One. 2021 Jul 2;16(7):e0254097. doi: 10.1371/journal.pone.0254097 (PMC8253411; doi:10.1371/journal.pone.0254097)
Supplement: S3 Table — (DOCX) [file pone.0254097.s003.docx]

**S3 Table. Conditional probability table for human-mediated colonization of northern pike in the Matanuska-Susitna basin, Alaska, USA.**

| **Input node** | | **State (Human-mediated colonization)** | | |
| --- | --- | --- | --- | --- |
| **AIR** | **ROAD** | **low** | **moderate** | **high** |
| none | close | 10 | 10 | 80 |
| none | moderate | 25 | 25 | 50 |
| none | far | 80 | 10 | 10 |
| low | close | 0 | 15 | 85 |
| low | moderate | 20 | 30 | 50 |
| low | far | 50 | 30 | 20 |
| moderate | close | 0 | 10 | 90 |
| moderate | moderate | 10 | 30 | 60 |
| moderate | far | 0 | 40 | 60 |
| high | close | 0 | 0 | 100 |
| high | moderate | 0 | 10 | 90 |
| high | far | 0 | 20 | 80 |
