# Supplementary material for: Vulnerability of Pacific salmon to invasion of northern pike (Esox lucius) in Southcentral Alaska
Source: PLoS One. 2021 Jul 2;16(7):e0254097. doi: 10.1371/journal.pone.0254097 (PMC8253411; doi:10.1371/journal.pone.0254097)
Supplement: S4 Table — (DOCX) [file pone.0254097.s004.docx]

**S4 Table. Conditional probability table for vulnerability of Pacific salmon in the Matanuska-Susitna basin, Alaska, USA.**

| **Input nodes** | | | **State (Vulnerability)** | | |
| --- | --- | --- | --- | --- | --- |
| **habitat** | **natural** | **human** | **low** | **moderate** | **high** |
| low | none | low | 90 | 5 | 5 |
| low | none | moderate | 85 | 10 | 5 |
| low | none | high | 80 | 15 | 5 |
| low | low | low | 85 | 10 | 5 |
| low | low | moderate | 80 | 15 | 5 |
| low | low | high | 75 | 20 | 5 |
| low | moderate | low | 70 | 25 | 5 |
| low | moderate | moderate | 65 | 30 | 5 |
| low | moderate | high | 60 | 35 | 5 |
| low | high | low | 55 | 40 | 5 |
| low | high | moderate | 50 | 45 | 5 |
| low | high | high | 45 | 50 | 5 |
| moderate | none | low | 55 | 40 | 5 |
| moderate | none | moderate | 50 | 45 | 5 |
| moderate | none | high | 45 | 50 | 5 |
| moderate | low | low | 50 | 45 | 5 |
| moderate | low | moderate | 45 | 50 | 5 |
| moderate | low | high | 40 | 55 | 5 |
| moderate | moderate | low | 30 | 60 | 10 |
| moderate | moderate | moderate | 25 | 50 | 25 |
| moderate | moderate | high | 20 | 55 | 25 |
| moderate | high | low | 10 | 60 | 30 |
| moderate | high | moderate | 5 | 45 | 50 |
| moderate | high | high | 5 | 40 | 55 |
| high | none | low | 10 | 60 | 30 |
| high | none | moderate | 5 | 45 | 50 |
| high | none | high | 5 | 40 | 55 |
| high | low | low | 5 | 45 | 50 |
| high | low | moderate | 5 | 40 | 55 |
| high | low | high | 5 | 35 | 60 |
| high | moderate | low | 5 | 25 | 70 |
| high | moderate | moderate | 5 | 20 | 75 |
| high | moderate | high | 0 | 20 | 80 |
| high | high | low | 0 | 10 | 90 |
| high | high | moderate | 0 | 5 | 95 |
| high | high | high | 0 | 0 | 100 |
