# Supplementary material for: Vulnerability of Pacific salmon to invasion of northern pike (Esox lucius) in Southcentral Alaska
Source: PLoS One. 2021 Jul 2;16(7):e0254097. doi: 10.1371/journal.pone.0254097 (PMC8253411; doi:10.1371/journal.pone.0254097)
Supplement: S5 Table — Ranks were weighted as follows: habitat overlap (Input node = habitat; high = 100), natural colonization (Input node = natural; high = 50), and human-mediated colonization (Input node = human; high = 10). The total possible weighted rank was 160, thus we used 80 as the inflection point for shifting vulnerability from low towards high. (DOCX) [file pone.0254097.s005.docx]

**S5 Table. Weighting for conditional probability table for vulnerability of Pacific salmon in the Matanuska-Susitna basin, Alaska, USA (S4 Table).**

| **Input nodes** | | | **Weighted ranks** | | | **State (Vulnerability)** | | | |
| --- | --- | --- | --- | --- | --- | --- | --- | --- | --- |
| **habitat** | **natural** | **human** | **habitat** | **natural** | **human** | **sum** | **low** | **moderate** | **high** |
| low | none | low | 0 | 0 | 0 | 0 | 90 | 5 | 5 |
| low | none | moderate | 0 | 0 | 5 | 5 | 85 | 10 | 5 |
| low | low | low | 0 | 5 | 0 | 5 | 85 | 10 | 5 |
| low | none | high | 0 | 0 | 10 | 10 | 80 | 15 | 5 |
| low | low | moderate | 0 | 5 | 5 | 10 | 80 | 15 | 5 |
| low | low | high | 0 | 5 | 10 | 15 | 75 | 20 | 5 |
| low | moderate | low | 0 | 25 | 0 | 25 | 70 | 25 | 5 |
| low | moderate | moderate | 0 | 25 | 5 | 30 | 65 | 30 | 5 |
| low | moderate | high | 0 | 25 | 10 | 35 | 60 | 35 | 5 |
| low | high | low | 0 | 50 | 0 | 50 | 55 | 40 | 5 |
| moderate | none | low | 50 | 0 | 0 | 50 | 55 | 40 | 5 |
| low | high | moderate | 0 | 50 | 5 | 55 | 50 | 45 | 5 |
| moderate | none | moderate | 50 | 0 | 5 | 55 | 50 | 45 | 5 |
| moderate | low | low | 50 | 5 | 0 | 55 | 50 | 45 | 5 |
| low | high | high | 0 | 50 | 10 | 60 | 45 | 50 | 5 |
| moderate | none | high | 50 | 0 | 10 | 60 | 45 | 50 | 5 |
| moderate | low | moderate | 50 | 5 | 5 | 60 | 45 | 50 | 5 |
| moderate | low | high | 50 | 5 | 10 | 65 | 40 | 55 | 5 |
| moderate | moderate | low | 50 | 25 | 0 | 75 | 30 | 60 | 10 |
| moderate | moderate | moderate | 50 | 25 | 5 | 80 | 25 | 50 | 25 |
| moderate | moderate | high | 50 | 25 | 10 | 85 | 20 | 55 | 25 |
| moderate | high | low | 50 | 50 | 0 | 100 | 10 | 60 | 30 |
| high | none | low | 100 | 0 | 0 | 100 | 10 | 60 | 30 |
| moderate | high | moderate | 50 | 50 | 5 | 105 | 5 | 45 | 50 |
| high | none | moderate | 100 | 0 | 5 | 105 | 5 | 45 | 50 |
| high | low | low | 100 | 5 | 0 | 105 | 5 | 45 | 50 |
| moderate | high | high | 50 | 50 | 10 | 110 | 5 | 40 | 55 |
| high | none | high | 100 | 0 | 10 | 110 | 5 | 40 | 55 |
| high | low | moderate | 100 | 5 | 5 | 110 | 5 | 40 | 55 |
| high | low | high | 100 | 5 | 10 | 115 | 5 | 35 | 60 |
| high | moderate | low | 100 | 25 | 0 | 125 | 5 | 25 | 70 |
| high | moderate | moderate | 100 | 25 | 5 | 130 | 5 | 20 | 75 |
| high | moderate | high | 100 | 25 | 10 | 135 | 0 | 20 | 80 |
| high | high | low | 100 | 50 | 0 | 150 | 0 | 10 | 90 |
| high | high | moderate | 100 | 50 | 5 | 155 | 0 | 5 | 95 |
| high | high | high | 100 | 50 | 10 | 160 | 0 | 0 | 100 |

Ranks were weighted as follows: habitat overlap (Input node = habitat; high = 100), natural colonization (Input node = natural; high = 50), and human-mediated colonization (Input node = human; high = 10). The total possible weighted rank was 160, thus we used 80 as the inflection point for shifting vulnerability from low towards high.
