# Supplementary material for: Vulnerability of Pacific salmon to invasion of northern pike (Esox lucius) in Southcentral Alaska
Source: PLoS One. 2021 Jul 2;16(7):e0254097. doi: 10.1371/journal.pone.0254097 (PMC8253411; doi:10.1371/journal.pone.0254097)
Supplement: S6 Table — Table represents Netica vulnerability class ‘moderate’. (DOCX) [file pone.0254097.s006.docx]

**S6 Table. Vulnerability of Pacific salmon by HUC-8 sub-basin in stream kilometers within the Matanuska-Susitna basin, Alaska, USA. Table represents Netica vulnerability class ‘moderate’.**

| **Sub-basin**  **(HUC-8)** | **Area**  **(km^2^)** | **Stream length**  **(km)** | **chum** | **coho** | **Chinook** | **pink** | **sockeye** |
| --- | --- | --- | --- | --- | --- | --- | --- |
| Anchorage | 3061 | 939 | 177 | 201 | 336 | 212 | 157 |
| Matanuska | 8662 | 2393 | 989 | 1142 | 1502 | 1189 | 788 |
| Upper Susitna River | 16346 | 5546 | 51 | 52 | 9 | 50 | 38 |
| Chulitna River | 6728 | 2280 | 67 | 66 | 30 | 75 | 53 |
| Talkeetna River | 5286 | 1681 | 53 | 48 | 53 | 63 | 44 |
| Yentna River | 15869 | 5988 | 443 | 485 | 485 | 400 | 262 |
| Lower Susitna River | 8855 | 4049 | 635 | 626 | 819 | 692 | 362 |
