# Supplementary material for: Vulnerability of Pacific salmon to invasion of northern pike (Esox lucius) in Southcentral Alaska
Source: PLoS One. 2021 Jul 2;16(7):e0254097. doi: 10.1371/journal.pone.0254097 (PMC8253411; doi:10.1371/journal.pone.0254097)
Supplement: S1 File — (DOCX) [file pone.0254097.s007.docx]

**S1 File. Creation of the landscape network**

The following steps were modified from the STARS user manual (available from: https://www.fs.fed.us/rm/boise/AWAE/projects/SSN_STARS/software_data.html#STARS) and performed by an ArcMap toolbox to create a landscape network (LSN) prior to creation of the spatial stream network (SSN). The R package *SSN* version 1.1.12 was used to calculate the pair-wise distance between reaches (‘sites’) and closest known source lakes (‘preds’).

1. The seven NetMap sub-basins were *Merged* and the resulting network pruned to reaches with an upstream area greater than 5 km^2^.
2. The stream network was *Dissolved* into a single polyline,
3. The endpoints of each reach identified using *Feature Vertices to Points >* *both_ends*
4. The network *split* at each point using the editor in ArcMap.
5. Stream flow direction was corrected to ensure downstream flow throughout the stream network using *Reverse Flow Direction*.
6. The resulting polyline was turned into a topologically corrected LSN by running the following in STARS:
   1. *Identify complex confluences*
   2. *Check network topology*
7. The centroid of all stream reaches, and invaded lakes were identified using *Feature to Point* and added to the SSN as ‘sites’ and ‘predictions’ (see STARS for details).
8. Five STARS tools were used to generate the spatial data necessary for conversion to the SSN.
   1. *Segment* *PI*
   2. *Additive* *Function – Edges*
   3. *Additive* *Function – Sites*
   4. *Upstream Distance – Edges*
   5. *Upstream Distance – Sites*
