# Supplementary material for: Vulnerability of Pacific salmon to invasion of northern pike (Esox lucius) in Southcentral Alaska
Source: PLoS One. 2021 Jul 2;16(7):e0254097. doi: 10.1371/journal.pone.0254097 (PMC8253411; doi:10.1371/journal.pone.0254097)
Supplement: S2 File — (DOCX) [file pone.0254097.s008.docx]

**S2 File. Calculation of lake fetch**

Due to the computational complexity of calculating fetch for thousands of lakes in the Matanuska-Susitna basin, a relationship between maximum lake length and lake fetch was derived and utilized as a proxy. Although not available for Alaska, the National Hydrography Database (NHDPlus Version 2) contains numerous lake metrics for 363,313 lakes in the contiguous United States. These data were used to parameterize a simple linear regression between lake fetch and maximum lake length (R^2^ = 0.916; P < 0.001),

F_i_ = 0.778*L_i_ + 32.1097 (1)

where *F_i_* represents fetch of lake *i* and *L_i_* represents the maximum length of lake *i*.

We calculated the maximum length of lakes (FType = 390; NetMap dataset) within the Matanuska-Susitna basin using the minimum bounding geography tool in ArcMap. The tool calculates the length and width of the smallest rectangle which fits around a polygon. We assumed the longest measure of the bounding rectangle to be representative of maximum lake length. Finally, we calculated lake fetch using the established relationship between maximum lake length and fetch (Equation 1), for all lakes.
